# Neuroimmune modulation following traumatic stress in rats: evidence for an immunoregulatory cascade mediated by c-Src, miRNA222 and PAK1

**DOI:** 10.1186/1742-2094-8-159

**Published:** 2011-11-14

**Authors:** Hui Zhao, Ranran Yao, Xiaoding Cao, Gencheng Wu

**Affiliations:** 1 Department of Integrative Medicine and Neurobiology, State Key Lab of Medical Neurobiology, Shanghai Medical College, Brain Research Institute, Fudan University, Shanghai, P. R. China

**Keywords:** c-Src, miRNA222, PAK-1, IL-1β, neuroimmune modulation

## Abstract

**Background:**

Neuroimmune modulation following traumatic stress is accompanied by cortical upregulation of c-Src expression, but the mechanistic details of the potential regulatory link between c-Src expression and immunosuppression have not been established.

**Methods:**

We used a combination of techniques to measure temporal changes in: (i) the parallel expression of c-Src and microRNA222; (ii) levels of PAK1 (p21-activated kinase 1); and (iii) the association between PAK1 and interleukin 1β signaling, both in cortex of rats following traumatic stress and in primary cortical neurons. Techniques included real-time PCR, immunoprecipitation, western blotting and subcellular fractionation by discontinuous centrifugation. We also measured lymphocyte proliferation and natural killer (NK) cell activity.

**Results:**

We confirm robust upregulation of c-Src expression following traumatic stress. c-Src upregulation was accompanied by marked increases in levels of miRNA222; other studied miRNAs were not affected by stress. We also established that PAK1 is a primary target for miRNA222, and that increased levels of miRNA222 following traumatic stress are accompanied by downregulation of PAK1 expression. PAK1 was shown to mediate the association of IL-1RI with lipid rafts and thereby enhance IL-1 signaling. Detailed analyses in cultured neurons and glial cells revealed that PAK1-mediated enhancement of IL-1RI activation is governed to a large extent by c-Src/miRNA222 signaling; this signaling played a central role in the modulation of lymphocyte proliferation and NK cell activity.

**Conclusions:**

Our results suggest that neuroimmune modulation following traumatic stress is mediated by a cascade that involves c-Src-mediated enhancement of miRNA222 expression and downregulation of PAK1, which in turn impairs signaling via IL-1β/IL1-RI, leading to immunosuppression. The regulatory networks involving c-Src/miRNA222 and PAK1/IL-1RI signaling have significant potential for the development of therapeutic approaches designed to promote recovery following traumatic injury.

## Background

Stress refers to the challenge, adversity, hardship, and affliction that organisms encounter in life, which jeopardize their physical and psychological well being [1]. A finely tuned spatiotemporal regulation of multiple events suggests hierarchic involvement of modulatory neurotransmitters and modified processes in pathways of gene expression that together could enable widely diverse stress responses [2,3]. For example, acetylcholine (ACh) acts as a stress response-regulating transmitter; and altered ACh levels are variously associated with changes in alternative splicing of pre-mRNA transcripts in brain neurons and peripheral blood cells [4]. Surgical trauma is one form of severe stress, which is associated with decreased splenocyte proliferation, reduced natural killer (NK) cell activity, and abnormal levels of several cytokines [5-7]. Importantly, neuroimmune modulation following surgical stress has been ascribed to molecular events taking place in cortical circuits. These can be separated into two stages - early events of immunosuppresion operating through an elaborate IL-1β pathway [8-13], and later progression marked by changes in c-Src signaling [14]. These dynamic alterations are likely to take place in distinct cellular compartments controlling the activation of different signaling cascades.

c-Src function is crucial for recovery from traumatic stress-mediated immunosuppression [14], but its mechanistic linkage to inflammation onset and progression remains to be elucidated. c-Src is a member of the Src family of protein kinases whose members play a crucial role in transducing extracellular signals to cytoplasmic and nuclear effectors, and thereby regulate a wide variety of cellular functions, including cell proliferation, differentiation and stress responses [15,16]. Functional overlap of c-Src and miRNA222 signaling has recently been demonstrated, and these factors are thought to play a joint regulatory role in tumor cell migration, nervous system development and neurodegenerative diseases [17]. However, the question of whether such signaling contributes to neuroimmune modulation in trauma remains to be clarified.

Of note, many microRNAs are involved in the neuroimmune pathway, which are named NeurimmiRs. Both peripheral and central immune insults have been shown to upregulate various NeurimmiRs, either in neurons, in surrounding cells (glia, microglia and infiltrating leukocytes) or in peripheral leukocytes. Owing to their physical properties and multiple roles in the nervous and immune systems, NeurimmiRs may initiate communication cascades via regulation of expression of numerous genes both in health and disease [18,19]. Besides reported NeurimmiRs, miRNA222 has been found to play critical roles in a variety of biological processes in the central nervous system (CNS), where p21-activated kinase 1 (PAK1) is one of its targets [20,21]. PAK1 upregulation in hippocampus and cortex is associated with stroke and neurite outgrowth, whereas downregulation of PAK1 has been recently reported in depression [22]. Further detailed studies have revealed that precise spatiotemporal expression of PAK1 proteins is required for the pleotropic effects of interleukin (IL)-1β [23] that require appropriate receptor expression and effective activation of intracellular signaling [23,24]. In the CNS, immune-like processes have been found to underlie responses not only to immune challenges but also to physiological and psychological stress. It has become evident that pro-inflammatory cytokines like IL-1β - which is produced predominantly by activated cells of the innate immune system such as monocytes, macrophages, and brain microglia - plays an important role in neuroendocrine and behavioral responses to various stresses [1,11]. Importantly, further research on IL-1β signaling has focused on phosphorylation and subcellular distribution of IL-1 receptor type I (IL-1RI) in lipid rafts, where these signaling pathways modulate IL-1β-induced cellular activation [25,26]. Together, these observations suggest that PAK1 could be a target for regulation mediated by c-Src and miRNA222 and thereby provide a mechanistic link between c-Src signaling and IL-1β activity following traumatic stress.

Notably, the prefrontal cortex (PFC) is known to play an important role in the integration of affective states with appropriate modulation of autonomic and neuroendocrine stress regulatory systems. There is evidence for manipulation of prefrontal cortical networks in conditions involving incorporation of adaptive behavior and prevention of excessive behavioral and physiological stress reactivity [27]. This may be especially true for traumatic stress-related c-Src and IL-1β signaling, which are enriched and initiated within this region [11,14]. Therefore, in the current study we sought to characterize molecular aspects of c-Src-related signaling in PFC which could modulate the onset or progression of immunosuppression induced by traumatic stress. It is well established that traumatic stress in rats leads to constitutive activation of neuroimmunomodulatory circuitry [28,29], and we have investigated the possibility that miRNA222 regulates a feedback loop that promotes immunosuppression induced by traumatic stress.

## Methods

### Traumatic animal model

All animal experiments were carried out in accordance with the guidelines and regulations for animal experimentation in NIH and Fudan University. SD adult male rats (Animal Center of Chinese Academy of Sciences, 200-250 g) were used in the current experiment. The animals were housed in groups (5 per cage) in a controlled environment on a 12 h light-dark cycle, and allowed to acclimate for a minimum of 5 days before conducting experiments. Water and food were available at all times.

Traumatic stress was performed as previously described [11]. Briefly, rats were anesthetized with pentobarbital sodium (35 mg/kg, i.p.), then were incised longitudinally to a length of 6 cm along the dorsal median line and 5 cm along the abdominal median line. After surgery, wounds were sutured and animals were kept warm in single housing, with care taken to keep sawdust bedding dry and clean. No post-operative infections occurred. The operation was performed 48 h after implanting a cannula, and tissue samples were taken 1, 3 and 7 days after the operation. Control rats were also anesthetized and underwent operation to implant a cannula.

### Intracerebroventricular injection of drugs

Implantation of the cannula was performed stereotaxically under anesthesia, a stainless steel guide cannula (0.5 mm in diameter) with an inserted cannula (0.25 mm in diameter) was implanted into right lateral ventricle (posterior 0.5, lateral 1.5, horizontal 4.5) and fixed onto the skull with dental cement. IL-1ra (10 units, Sigma Aldrich, St. Louis, MO), PAK1 antibody (10 μg), and recombinant adenovirus (5 × 10^9 ^plaque-forming units (pfu)) dissolved in sterilized PBS were injected over 10s via the cannula in a volume of 10 μl. Rats from the control group were injected with vehicle. At the end of each procudure, the entire injector system was left in place for an additional 10 min to minimize reflux. The position of the cannula was assessed by histological examination, and data were collected from experiments in which correct insertion of the cannula was verified. Animals were operated upon and killed 24 h after IL-1ra and PAK1 antibody injection, or 72 h after recombinant adenovirus injection.

### Recombinant adenoviruses

cDNA for dominant-negative (K296R/Y528F, DN c-Src), or constitutively active (Y528F, CA c-Src) forms (Upstate Biotechnology, Lake Placid, NY) were cloned into adenoviral shuttle vector pDE1sp1A (Microbix Biosystems, Inc. Canada). After homologous recombination in vivo with the backbone vector PJM17, plaques resulting from viral cytopathic effects were selected and expanded in 293 cells. Positive plaques were further purified and large-scale production of adenovirus was carried out by two sequential CsCl gradients and PD-10 Sephadex chromatography.

### Immunofluorescent analysis

Rats were anesthetized with sodium pentobarbital (35 mg/kb, i.p.) and perfused transcardially with fixative (4% paraformaldehyde). Coronal brain sections (25 μm) were obtained using a cryostat. Frozen sections were subjected to immunostaining with anti-PAK1 at 1:200 or anti-c-Src at 1:100 (Upstate Biotechnology, Lake Placid, NY), then transferred into Alexa594 conjugated anti-rabbit antiserum (1:1000, Invitrogen, Carlsbad, CA) for 1 h. Data derived from each group were analyzed by Leika Q500IW image analysis system. Frontal cortex was chosen for analysis and immunopositive cells were semi-quantified using photomicrography.

For cell immunofluorescent staining, neuronal or glial cells were dissociated and plated into covers lips pretreated with 0.1% polyethylenemine. After 10 days of growth, the coverslips were subjected to anti-c-Src- and Alexa488-conjugated secondary antibodies, and anti-PAK1- and Alexa594-conjugated antibodies subsequently. Data were analyzed using a Leika Q500IW image analysis system.

### Immunological assay

For lymphocyte proliferation, spleens were pressed through stainless steel mesh and red blood cells were lysed by treatment with NH_4_Cl solution. Cell were suspended at 1 × 10^7 ^cells/ml in a final volume of 200 μl of complete tissue culture medium (RPMI 1640 supplemented with 10% heat activated fetal calf serum, 2 mM L-glutamine), and seeded in triplicate in U-bottom 96-well plates in the presence or absence of concanavalin A (Con A, 1 mg/L, Sigma Aldrich, St. Louis, MO). Plates were incubated at 37°C in a 5% CO_2_. After 48 h, cultures were labeled with 0.5 μCi of [^3^H] thymidine (Amersham Biosciences, Piscataway, NY). Cells were harvested using a cell harvester 24 h later. Samples were counted in a liquid scintillation counter. Proliferation results are presented as mean cpm ± SD of triplicate cultures in 5 animals.

For natural killer cell cytotoxicity, suspensions of YAC-1 lymphoma cells, with a concentration of 2 × 10^5^/ml at a final volume of 100 μl, were targeted with 0.5 μCi of [^3^H] thymidine and incubated at 37°C, 5% CO_2 _for 6 h. Then, the spleens were homogenized and the resultant cell suspensions pooled in the presence or absence of Con A and seeded in triplicate with effector:target ratios of 50:1 for 16 h. Cytotoxic activity results were determined as follows:

$$\begin{array}{l} \text{Percent response}=[(\text{counts in tested well-counts in spontaneous response well})/\\ (\text{counts in maximum response well-counts in spontaneous response well})]\times \text{1}00 \end{array}$$

### IL-1RI Production

IL-1RI expression was measured using an ELISA kit (R&D systems, Minneapolis, MN). Briefly, frontal cortex was collected and suspended in equal volumes of 50 μl diluent buffer. Plates were incubated for 2 h at 37°C. Hybridization reactions were stopped by several washes and the plates were subsequently incubated with biotinylated anti-IL-1RI solution for 1 h, streptavidin-HRP solution for 30 min, and with the stabilized chromogen for 30 min. Stop solution (100 μl) was added to each well and the optical density was measured at 450 nm using BioRad microreader (Hayward, CA). Data were normalized, and expressed as mean ± SD from 5 animals, each performed in triplicate.

### TaqMan reverse transcription (RT)-PCR for miRNA quantification

Total RNA was isolated from frontal cortex (50 mg) or cortical neurons (1 × 10^6^) with Trizol™ (Invitrogen, Carlsbad, VA) according to manufacturer's protocol. MicroRNA 218, 224, 142, 222, 126, 296, 194, 206 quantification was carried out by reverse transcribing total RNA using Taqman™ microRNA reverse transcription kit and subjected to real-time PCR using TaqMan™ MicroRNA Assay kit (Applied Biosystems, Carlsbad, CA). Reactions were performed using Stratagene Mx3000 instrument in triplicate. Real-time PR data was analyzed using a ΔΔCt calculation. A p value of less than 0.05, when considering treated animals or cells *vs*. control group, was considered significant.

### Primary neuron culture and treatment

For primary neuron cultures, rat fetuses were removed from pregnant rats on embryonic day 18. Cortices were dissected and collected in Hanks' balanced salt solution. Cells were dissociated and plated at a density of 10^6 ^cells per well into 24-well tissue culture plates pretreated with 0.1% polyethylenemine. Cells were maintained in serum-free Neurobasal medium containing B27 supplement (Gibco, Rockville, MD). After 3-4 days in culture, neurons sent out long processes. By 10 days, flow cytometry showed that MAP_2 _immunopositive cells accounted for more than 95% of cells, and the indicated treatments were performed at this same time.

c-Src plasmid, microRNA222 mimetic and microRNA222 inhibitor (Dharmacon RNA Technologies, Lafayette, CO) were transfected into primary neurons using Lipofectamine 2000 according to the manufacturer's instructions (Qiagen, Valencia, CA). In brief, 1 × 10^6 ^neurons were transfected with 10 pmol of microRNA222 mimic and microRNA222 inhibitor. Following transfection, neurons were cultured for another 48 h prior to experiments.

For experiments using IL-1β (R&D systems, Minneapolis, MN. 20 ng/ml, 24 h), IL-1ra (10 ng/ml, 24 h), PP2 (5 μM, 30 min, Tocris Bioscience, Ellisville, MO) was added to the culture medium for the indicated time periods and followed by analysis.

### Detergent-free preparation of lipid rafts

The isolation of lipid rafts in the current study was adapted from Lisanti's lab [30]. Tissues/cells were homogenized in 2 ml of 500 mM sodium carbonate, PH11.0. Homogenization was carried out sequentially in the following order using a loose-fitting Dounce homogenizer (10 strokes), three 10 s bursts of a Polytron tissue grinder (Brinkmann Instruments, Inc., Westbury, NY) at setting 6, followed by one 30 s burst at setting 4 and one 30 s burst at a setting 8 of a sonicator equipped with a micro-probe (Heat systems-Ultrasonics, Inc., Plainview, NY). The homogenate was then adjusted to 45% sucrose by the addition of 2 ml of 90% sucrose prepared in MES-buffered saline (MBS) at pH 6.8 and placed at the bottom of an ultracentrifuge tube. The lysate was then overlaid with 4 ml of 35% sucrose and 4 ml of 5% sucrose, both prepared in MBS containing 250 mM sodium carbonate at pH 11. The discontinuous gradient was centrifuged at 39,000 rpm for 16-20 h in a SW41 rotor. Light-scattering layers at the 5-35% and 35-45% sucrose interfaces were collected and referred to as raft (GM-1 positive) and non-raft fractions; proteins were then analyzed by western blot.

### Immunoprecipitation and western blot

Frontal cortex was sonicated with about seven volumes of protein-extraction buffer containing 20 mM HEPES (pH 7.5), 10 mM potassium chloride, 1.5 mM magnesium chloride, 1 mM ethylenediaminetetraacetic acid, 1 mM EGTA, and 1× Complete Protease Inhibitor (Roche Applied Science). The sonicated sample was centrifuged at 10,000 g for 15 min at 4°C, and the supernatant was incubated with anti-IL-1RI (1:200; R&D systems, Minneapolis, MN) on a rotating platform overnight, followed by incubation with 20 μl protein G agarose beads (Pierce Biotechnology) for 2 h at 4°C. The beads were washed three times in lysis buffer, and proteins were extracted and resolved in SDS-polyacrylamide gels, and transferred to polyvinylidene difluoride membranes (PVDF, Amersham). The membranes were probed with anti-PAK1 (1:1000), and subsequent alkaline phosphatase-conjugated secondary antibody (1:5000; Amersham Biosciences, Piscataway, NJ). Bands were detected by ECF substrate (Amersham Biosciences, Piscataway, NJ) and were quantified using ImageQquant software.

### Statistics

Data are represented as mean ± SD and analyzed with Prism 5 software. For all data sets, normality and homocedasticity assumptions were reached, validating the application of one-way ANOVA, followed by Dunnett test as post hoc test to do comparisons. Differences were considered significant for p < 0.05.

## Results

### Induction of c-Src signaling cascades by traumatic stress

**Figure 1:**
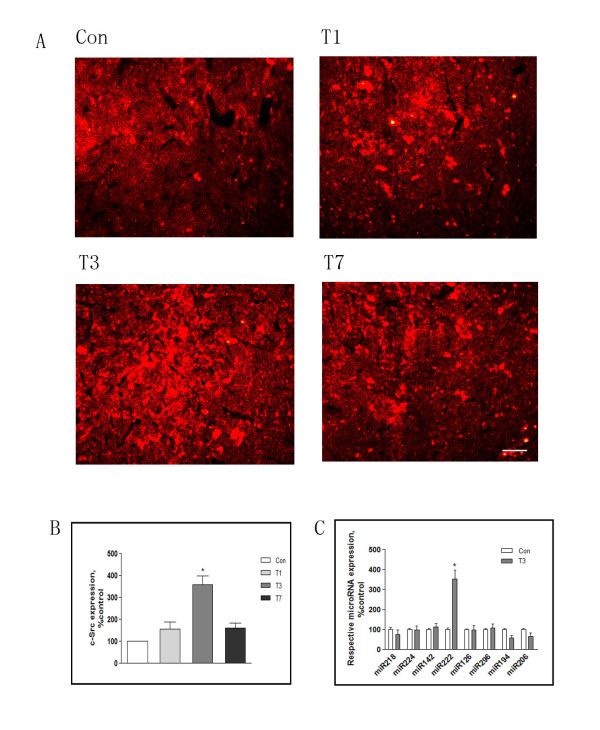
**Induction of c-Src signaling cascades by traumatic stress**. Rats were killed 1, 3, or 7 days after traumatic stress (n = 5 for each time point). Cross sections of frontal cortex were immunostained with anti-c-Src antibody (A), and the density of immunopositive cells was semi-quantified in three randomly chosen areas (B). Real-time PCR was used to analyze microRNAs in frontal cortex (C). Con: control; T1, 3, 7: 1, 3, 7 days after trauma. Data are presented as percentage of control. Values represent mean ± SD for 3 independent experiments. *P <0.05 *vs *Con. Scale bars = 50 μm.

We first examined c-Src expression in the frontal cortex following traumatic stress. Rats were challenged with surgical trauma and analysis was performed at days 1, 3 and 7 after trauma-timepoints defined by our previous observations [11]. Immunofluorescence revealed that c-Src immunopositivity was increased in frontal cortex, reaching a maximum at 3 days following trauma. Interestingly, fluorescence progressively decreased thereafter, returning to control levels at 7 days (Figure 1A, B).

Eight miRNAs have been reported to be regulated by c-Src [31]. Real-time PCR revealed that levels of miRNA222 in frontal cortex were robustly increased at day 3 following trauma, a timepoint corresponding to maximum upregulation of c-Src. Seven other miRNAs were also examined: miRNAs 218, 194, 206 showed weakened signals after traumatic stress whereas there were no detectable changes in the levels of miRNAs 224, 142, 126, or 296. It is therefore possible that miRNA222 upregulation is associated with c-Src signaling and that this could contribute to recovery from immunosuppression following traumatic stress (Figure 1C).

### PAK1 is a miRNA222 target

**Figure 2:**
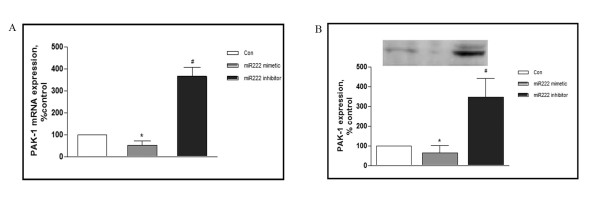
**PAK1 is a miRNA222 target**. Rat cortical neurons were transfected with control RNA, microRNA222 mimetic, or microRNA222 inhibitor using Lipofectamine 2000. Two days after transfection, mRNA (A) and protein (B) levels of PAK1 were determined by real-time PCR and western blot, respectively. Results are normalized against an internal control (β-actin) and further normalized against the results obtained from cultures transfected with control RNA. The graph depicts percentage expression under the indicated treatments, relative to controls. Data were analyzed by one-way ANOVA with Dunnett test as a post hoc test for the comparisons.* *P <*0.05 *vs *Con, # *P <*0.05 *vs *microRNA222 mimetic.

To address whether PAK1 is a target for miRNA222, a miRNA222 mimetic and/or a miRNA222 inhibitor were transfected into primary cultured cortical neurons. As shown in Figure 2A and 2B, the miRNA222 mimetic decreased mRNA levels for PAK1; conversely, the miRNA222 inhibitor increased the levels of PAK1 mRNA. Similar effects were observed at the protein level: expression of PAK1 polypeptide was decreased by the miRNA222 mimetic whereas the miRNA222 inhibitor increased PAK1 protein levels. We conclude that PAK1 is negatively regulated by miRNA222.

### Time-dependent PAK1 expression in response to traumatic stress

**Figure 3:**
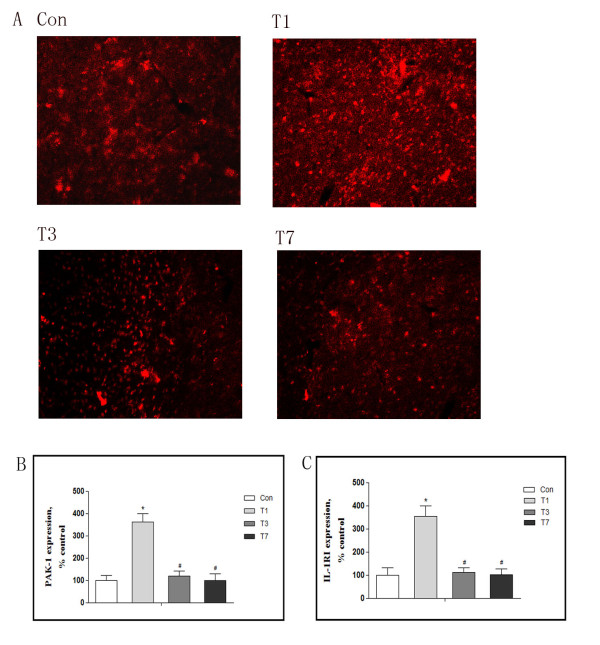
**Time-dependent PAK1 expression in response to traumatic stress**. Rats were killed 1, 3, or 7 days after traumatic stress (n = 5 for each time point). Cross sections of frontal cortex were immunostained for anti-PAK1 antibody (A), and the density of immunopositive cells was semi-quantified in three randomly chosen areas (B). Frontal cortex homogenates were prepared, and IL-1RI expression was determined by ELISA assay (C). Con: control; T1, 3, 7: 1, 3, 7 days after trauma. Data are presented as percentage of control; each value represents mean ± SD for three independent experiments. *P <0.05 *vs *Con. Scale bars = 50 μm.

Immunofluorescence using anti-PAK1 antibody on sections of frontal cortex demonstrated a time-dependent modulation of protein levels following trauma. Levels were strongly increased by 1 day after trauma, but decreased progressively at days 3 and 7 (Figure 3A).

Because PAK1 is known to modulate the cellular effects of IL-1β, we investigated if changes in PAK1 expression are accompanied by parallel changes in expression of the IL-1 receptor IL-1RI. As shown in Figure 3B, ELISA assay revealed that IL-1RI expression was increased by over 3-fold 1 day after trauma (354.0 ± 45.7% control), and gradually decreased thereafter, returning to control levels at day 7 (Figure 3B). The pattern of PAK1 expression paralleled that previously reported for IL-1β signaling after trauma [11], suggesting a potential association with neuroimmune modulation in the traumatic rat.

### PAK1 and IL-1RI modulation following traumatic stress

**Figure 4:**
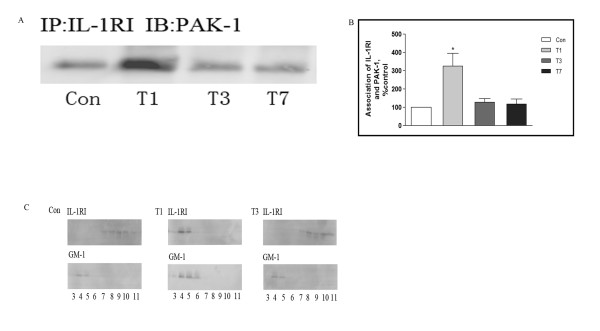
**PAK1 and IL-1RI modulation following traumatic stress**. Rats were killed 1, 3, or 7 days after traumatic stress (n = 5 for each time point), and frontal cortex homogenates were prepared. Immunoprecipitation was used to analyze alterations of PAK1 and IL-RI interaction. The immunoprecipitation antibody was anti-IL-1RI and the immunoblotting antibody was anti-PAK1 (A). Panel B depicts quantitative analysis of A. Data are presented as percentage of control, with the density of PAK1 in the control group (without operation) set at 100%. Values represent mean ± SD for 3 independent experiments. *P <0.05 *vs *Con. A lipid raft preparation was prepared to determine subcellular distribution of IL-1RI. Western blot analysis was used to detect IL-1RI expression in fractions 3-11, and GM-1 immunopositive fractions were identified as lipid raft fractions (C). Con: control; T1, 3: 1, 3 days after trauma.

It has been previously reported that PAK1 can interact directly with IL-1RI [25,26]. We therefore investigated whether the interaction is altered following traumatic stress. As shown in Figure 4A, B, anti-IL-1RI immunoprecipitates of rat cortex following trauma were significantly enriched in PAK1 material; the binding interaction was highest at day 1 following trauma and declined progressively thereafter. This result suggests that trauma augments the interaction between PAK1 and IL-1R1.

To address whether the increased binding is accompanied by changes in the cellular distribution of IL-1RI, subcellular fractions from prefrontal cortex were analyzed by western blotting for IL-1RI. As shown in Figure 4C, monosialoganglioside GM-1, a marker of lipid rafts, was highly enriched in fractions 4 and 5, indicating that these represent the lipid-raft membrane microdomain. In control rats, the IL-1RI immunopositive signal was generally present in the non-raft fractions. However, at day 1 following trauma IL-1RI was redistributed, and immunoreactivity was predominantly associated with lipid-raft fractions 4 and 5. The proportion of IL-1RI associated with the raft fraction then declined, and by days 3 and 7 following trauma IL-1RI immunopositivity was widely distributed in non-raft fractions (Figure 4C). IL-1RI activation is known to be accompanied by phosphorylation and recruitment into lipid rafts. In addition to stress induction of IL-1RI expression, our data are consistent with the possibility that elevated levels of PAK1 following traumatic stress also lead to redistribution and/or activation of receptor, thereby increasing the cellular effects of IL-1β.

### Modulation of PAK-1 signaling in cultured neurons and glial cells

**Figure 5:**
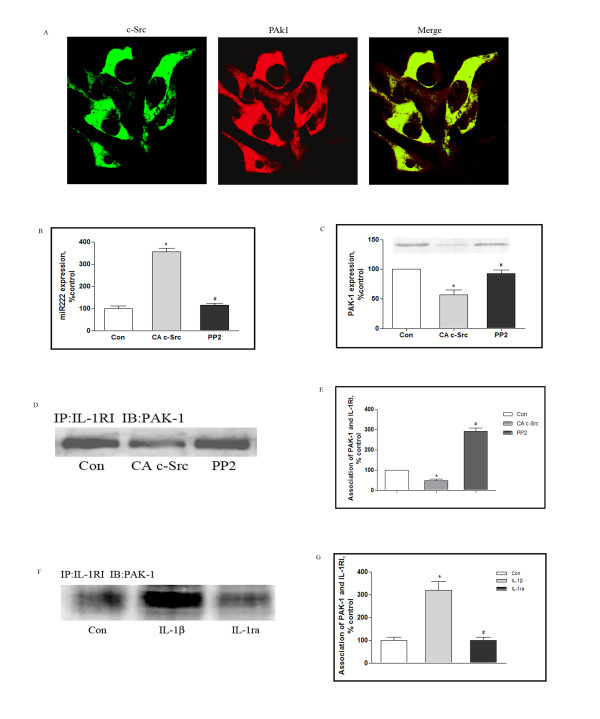
**Modulation of PAK-1 signaling in cultured neurons**. Rat cortical neurons were grown on coverslips for 10 days. Neurons were then immunostained using anti-c-Src and anti-PAK1 antibodies, and double-labeled cells were identified using a Leika Q500IW image analysis system (A). Neurons were treated with vehicle or PP2 for 30 min, and then assessed for microRNA222 (B) and PAK 1 (C) expression using real-time PCR and western blot, respectively. (D) Directed expression of c-Src in cultured neurons by transfection with adenovirus expressing active c-Src (CA c-Src). Endogenous c-Src activity was blocked using the synthetic inhibitor PP2, and association of PAK1 with IL-1RI was determined by immunoprecipitation assay. The immunoprecipitation antibody was anti-IL-1RI and the immunoblotting antibody was anti-PAK1. (E) The graph depicts expressions as percentages of controls. (F) Neurons were exposed to IL-1β and IL-1ra as described in Methods, and immunoprecipitation was used to analyze the association between PAK1 and IL-RI. The immunoprecipitation antibody was anti-IL-1RI and the immunoblotting antibody was anti-PAK1. The graph depicts expressions as percentages of controls (G). The results were normalized against an internal control and further normalized against the results obtained from control cultures. Data were analyzed by one-way ANOVA with Dunnett test as a post hoc test to assess comparisons.* *P <*0.05 *vs *Con, # *P <*0.05 *v*s c-Src, or IL-1β. Scale bars = 50 μm.

Neuronal and glial cells cohabit the CNS and both cell types demonstrate marked changes associated with neuroimmune modulation following traumatic stress [32]. We therefore examined the cellular distribution and levels of c-Src and PAK1 in neuronal and glial cells in culture. As shown in Figure 5A, immunostaining for c-Src (green fluorescence) and PAK1 (red fluorescence) revealed widespread dual staining in neurons (yellow coloration), suggesting that c-Src and PAK1 are largely colocalized in these cells. The same experiment was repeated for astrocytes and microglia, and overlap of c-Src and PAK1 fluorescence was also observed in these cells (data not presented). This result argues that coexpression of the two proteins is likely to be widespread in the CNS.

We then examined whether c-Src overexpression can modulate the expression of PAK1. As shown in Figure 5D, E, directed expression of c-Src in cultured neurons by transfection with adenovirus expressing constitutively active c-Src (CA c-Src) led to a marked reduction in levels of PAK1 expression and, moreover, upregulated levels of miRNA222. Conversely, when endogenous c-Src activity was blocked by the synthetic inhibitor PP2, miRNA222 expression was downregulated and PAK1 expression was strengthened (Figure 5B, C). In the meantime, the association of PAK1 and IL-1RI was affected by c-Src modulation, which was decreased by CA c-Src (48 ± 7% control) and elevated by PP2 (291 ± 16% control) (Figure 5D, E). Moreover, when neurons were exposed to IL-1β, the association of PAK1 with IL-1RI was dramatically enhanced compared to cells in the absence of IL-1β, and this effect was potently and specifically blocked by inhibition of IL-1RI by IL-1ra (Figure 5F, G). Equivalent results were obtained in cultured astrocytes and microglia (data not shown).

### c-Src signaling in neuroimmune modulation in the trauma rat

These observations together suggest that c-Src is strongly upregulated by traumatic stress, and is moreover a potent regulator of miRNA222 and PAK1: it is therefore possible that changes in the levels of PAK1 following stress could in turn could be responsible for alterations in IL-1RI receptor activation following trauma. We therefore addressed whether modulation of c-Src activity *in vivo *would impact upon the expression of miRNA222 and PAK1 and on the PAK1 interaction with IL-1RI.

**Figure 6:**
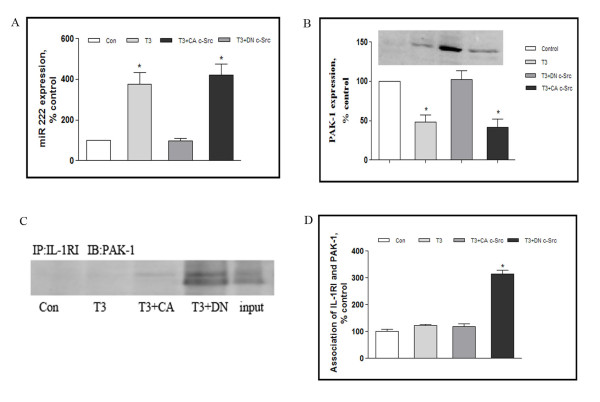
**PAK1 signaling modulation in traumatized rats**. Rats were subjected to surgical trauma, and 3 days later some of these rats were injected icv with adenovirus expressing either the dominant-negative (DN) form of c-Src or the equivalent form of constitutively active (CA) c-Src. Thus, 4 groups of rats were created: Controls (rats with no trauma), T3 (rats killed 3 days after trauma), T3+DN c-Src (rats treated with DN c-Src 3 days after trauma and killed 72 hours later), and T3+CA c-Src (rats treated with CA c-Src 3 days after trauma and killed 72 hours later) (n = 5 for each group). Homogenates of frontal cortex were prepared and assessed for microRNA222 (A) and PAK1 (B) expression using real-time PCR and western blot, respectively. The interaction of PAK1 and IL-RI was assessed by immunoprecipitation (C, D). Data are presented as percentage of control. Values represent mean ± SD for 3 independent experiments. *P <0.05 *vs *Con. Con: control; T3: 3 days after trauma.

Accordingly, at day 3 following trauma rats were injected icv with adenovirus expressing the dominant-negative (DN) form of c-Src, and changes in levels of miRNA222 and PAK1 were measured 72 hour later. As shown in Figure 6A, B, DN-c-Src resulted in a dramatic reduction in levels of miRNA222 and an equally robust increase in levels of PAK1 expression. We also explored the effects of administering the equivalent form of constitutively active (CA) c-Src. CA-c-Src administration resulted in an inverse effect, leading to increased miRNA222 levels and decreased PAK1 expression. Furthermore, the association of PAK1 and IL-1RI was also similarly modulated by administration of DN-c-SRc or CA-c-Src (Figure 6C, D).

**Figure 7:**
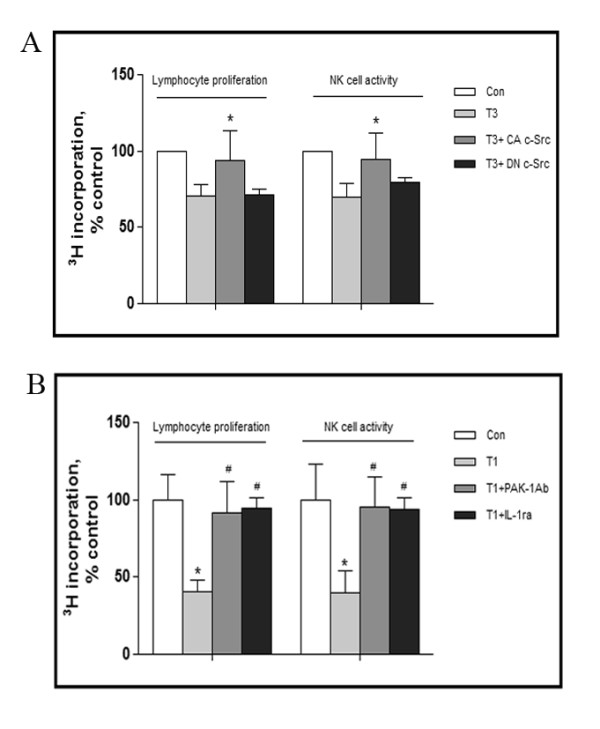
**c-Src signaling in neuroimmune modulation in traumatized rats**. Four groups of rats were prepared as described in Fig. 6: Con, T3, T3+DN c-Src, T3+CA c-Src (n = 5 for each group). Homogenates of spleen were prepared, and lymphocyte proliferation and NK cell activity were assayed by [^3^H] incorporation (A). Rats were subjected to surgical trauma, and 1 day later some of these rats were treated with PAK-1 Ab or IL-1ra. Thus, 4 groups of rats were created: Controls (Con; no trauma), T1 (rats killed 1 day after surgical trauma), T1+PAK-1 Ab (icv administration of antibody against PAK1, 1 day after trauma and killed 24 hours later), and T1+IL-1ra (icv administration of antibody against IL-1ra 1 day after trauma and killed 24 hours later) (n = 5 for each group). Homogenates of spleen were collected, and lymphocyte proliferation and NK cell activity were assayed by [^3^H] incorporation (B). Data are presented as percentage of control. Values represent mean ± SD for 3 independent experiments. *p < 0.05 *vs *Con, #p < 0.05 *vs *traumatized.

These data argue that c-Src is a positive regulator of miRNA222. Because PAK1 is a target for miRNA222, it is possible that c-Src modulates the PAK1-IL-1RI interaction by upregulating miRNA222 and inhibiting the expression of PAK1. Given that c-Src is strongly upregulated by traumatic stress, miRNA222 is a strong contender for the mechanistic link between c-Src activation and neuroimmune modulation following traumatic stress. To address this possibility we studied the effects of c-Src modulation on the suppression of lymphocyte proliferation and NK cell activity following traumatic stress. This revealed that inhibition of c-Src by DN c-Src led to a significant reduction of both lymphocyte proliferations and NK cell activity, [^3^H] incorporation for lymphocyte proliferation was 71 ± 8 and 71 ± 9% of control at day 3 after trauma and DN c-Src injection respectively. For NK cell activity, they were 70 ± 9 and 79 ± 7% of control, whereas, conversely, c-Src activation promoted the recovery from immunosuppression (Figure 7A).

To investigate the potential involvement of PAK1 in this process, antibody against PAK1 was injected icv. As shown in Figure 7B, abrogation of PAK1 activity with anti-PAK1 decreased lymphocyte proliferation and NK cell activity. We attribute this effect to inhibition of PAK1 enhancement of IL-1RI receptor expression and activation. To confirm that IL-1RI plays a role in this system, we investigated the effects of administering IL-1ra. As also shown in Figure 7B, IL-1ra exerted a similar progressive effect on PAK1 in the traumatic rat.

## Discussion

Recently, it has been reported that c-Src is likely to play a regulatory role in immunosuppression induced by trauma in rats [14]. In the present paper we have shown that activation of c-Src is accompanied by strong upregulation of expression of miRNA222, an inhibitor of the immunoregulator PAK1. We therefore postulate that miRNA222 provides a mechanistic link between c-Src and immunosuppression following traumatic stress.

Members of the Src family of protein tyrosine kinases are known to mediate a signaling cascade that relays information from the cell surface to the nucleus, promoting an array of cellular responses [14,33]. Tyrosine-phosphorylated signaling molecules have been directly implicated in neurite outgrowth that is thought to reflect an early step in neuronal regeneration [34-36]. Recent studies have revealed that c-Src overlaps functionally with miRNA222, and their combined effects modulate not only cancer cell proliferation [37] but also the fine tuning of gene expression during cell differentiation and brain development [38]. The interaction between c-Src and miRNA222 expression reported here is therefore likely to play a role in orchestrating neuroimmune changes following traumatic stress.

miRNAs are small noncoding RNAs that typically bind to the 3'-untranslated regions of protein-coding genes, repressing their expression by translational inhibition and/or promoting mRNA degradation [39]. PAK1 has been identified as one of the targets of miRNA222 [40,41], a finding confirmed here. Extending this work, we demonstrate that, following traumatic stress, miRNA222 and PAK1 expression in frontal cortex are inversely modulated. Specifically, PAK1 expression is strongly upregulated at day 1 following trauma, and thereafter progressively decreases to control levels at day 7. These observations suggest the hypothesis that increased miRNA levels induced by traumatic stress exert their cellular effects by inhibiting PAK1 expression, and thereby downregulating PAK1-mediated facilitation of IL-1 signaling and, potentially, mediating recovery from immunosuppression following trauma.

IL-1β was the first cytokine to be associated with modulation of neuroendocrine systems, particularly the hypothalamic pituitary-adrenal axis (HPA) and the hypothalamic-pituitary-gonadal axis, in the 1980s. To date, IL-1β has remained the most studied inflammatory cytokine in the mediation of immunological and psychological stress responses. IL-1β signaling is mediated by a complex system, and IL-1 receptor type I (IL-1RI) appears to mediate all of the known biological functions of IL-1 [1]. In regard to traumatic stress, we have found that elevated IL-1β expression is a primary response to traumatic stress [11], and that activation of IL-1β signaling is postulated to mediate immunosuppression in the traumatic rat [11]. In the present study we report that IL-1RI expression is dramatically increased at day 1 following trauma. Importantly, upregulation was accompanied by both increased PAK1 binding and accumulation of IL-1RI in lipid-raft fractions, a marker of receptor activation. Detailed examination of the role of PAK1 indicated that activated PAK1 is enriched primarily in postmitotic neurons and major axonal tracts [42,43]. PAK1 activation reflects its recruitment to the plasma membrane, and this has been demonstrated both in post-traumatic disorders and in progressive neurodegenerative conditions [44]. It was also recently reported that PAK1 activation could be responsible for changes in IL-1β/IL-RI activation in the CNS following stress [45-48]. It is therefore possible that PAK1 modulates IL-1RI activation and translocation into lipid rafts, thereby fine-tuning IL-1β signaling in the onset and progression of immunosuppression in the traumatic rat.

It is noteworthy that these signaling cascades also appear to operate in cultured neuron and glial cells. PAK1 is a target for miRNA222, and c-Src reproducibly produced up- or downregulation of miRNA222 and PAK1. PAK1 could function as a conveying molecule to enhance phosphorylation and lipid-raft association of IL-1RI, thereby facilitating IL-1β signaling. Importantly, c-Src activation is likely to modulate the association of PAK1 and IL-1RI. We therefore propose that c-Src upregulation following traumatic stress exerts its neuroimmunomodulatory effects by upregulating miRNA222, thereby reducing PAK1 expression and downregulating IL1-β/IL1-RI signaling.

Certainly, there are many NeurimmiRs, which primarily enable modulation of both immune and stress responses through direct or indirect alterations of neuron-glia signaling. Based on the present observations, miRNA222 may act as a "negotiator" between c-Src and IL-1β signaling compartments [18]. Also, miRNA222 is likely to have other targets in addition to PAK1, and the impact of c-Src modulation of miRNA222 expression may not be restricted to PAK1. However, the observation that the time course of miRNA222 overexpression following traumatic stress matches both the depression of PAK1 expression and immunosuppression supports the contention that PAK1 is a primary target for miRNA222 and contributes to neuroimmune modulation following traumatic stress.

## Conclusions

In summary, we report that c-Src activation following traumatic stress leads to a robust increase in levels of miRNA222 and a corresponding decrease in expression of the neuromodulator PAK1, a confirmed target for miRNA222. PAK1 is a key regulator of IL-1β signaling through its association with IL-1RI and, moreover, modulates the subcellular distribution of IL-1RI, enhancing its association with lipid rafts and its signaling activity. Together our data suggest that the pronounced immunosuppression that occurs in the CNS following traumatic stress [47,48] is mediated by a regulatory cascade involving c-Src, miRNA22, and PAK1 that then leads to abrogation of IL-1RI signaling.

## Abbreviations

Icv: intracerebraventricular injection; IL-1β: interleukin-1β; IL-1RI: type 1 IL-1 receptor; MBS: MES-buffered saline (MES: 2-[morpholino]ethanesulfonic acid); NK cell: natural killer cell; PAK1: Rac1/p21-activated kinase 1; PP2: 4-amino-5-(4-chlorophenyl)-7-(t-butyl)pyrazolo[3,4-d]pyrimidine).

## Competing interests

The authors declare that they have no competing interests.

## Authors' contributions

HZ produced the hypothesis for this study. XC and GW gave extensive advice on the study. RY was responsible for the animal and primary neuron and glial cell culture study. All authors read the manuscript, studied it critically for its intellectual content and approved the final draft.
